# The role of urbanization in soil and groundwater contamination by heavy metals and pathogenic bacteria: A case study from Oman

**DOI:** 10.1016/j.heliyon.2019.e01771

**Published:** 2019-05-27

**Authors:** Baby Shaharoona, Said Al-Ismaily, Ahmed Al-Mayahi, Nadhira Al-Harrasi, Ruqaiya Al-Kindi, Abdullah Al-Sulaimi, Hamad Al-Busaidi, Mohammed Al-Abri

**Affiliations:** a Department of Soils, Water, and Agricultural Engineering, Sultan Qaboos University, Oman; b Department of Animal and Veterinary Sciences; Sultan Qaboos University, Oman

**Keywords:** Environmental science

## Abstract

This study assessed the perception of urban residents of A'Seeb city, Oman, about the impact of their activities on environment. A sociological survey using questionnaire was used to know the residents' perceptions about urban gardening, municipal-waste disposal, and soil and water contamination. Viable pathogenic bacteria, water soluble metals, basic cations, salinity, and texture were quantified and identified in soil and groundwater in proximity of urban gardens and municipal-waste disposal sites. The majority of surveyed residents are not paying attention to the negative consequences of their activities on soil and environment. Although the measured heavy metals concentrations in some of the contaminated sites were significant but still below the international standards. Fecal contaminants reported in in some samples from gardens, garbage-disposal sites and groundwater. Human pathogens belonging to risk group-2 including *Klebsiella pneumonia, Shigella spp* and *E. Coil* were identified. More socio-environmental studies required to correlate the behavior of urban residents and pollution and to delineate the sources of the detected pathogenic bacteria. Our results set a foundation for future studies on urban soils and associated residence behaviors and practices in Oman and the neighboring Gulf countries.

## 1 Introduction

The urban population is rapidly growing all over the world and more than half of the world population (54%) inhabits in the urban neighborhoods. This rising trend of urbanization is expected to continue and could reach up to 60% in the year 2030 (United Nations, 2016). The surge in urbanization is resulting in higher population densities in the cities which in turn is threatening sustainable development and creating many environmental and social problems (Mutatkar, 1995; McMichael, 2000; Henderson, 2002; Da-Gang et al., 2008). Many of the developing countries are facing more problems of higher metropolitan agglomerations as the pace of urbanization is faster in developing nations which is gravely surpassing the capacities of these cities to provide adequate facilities to the urban inhabitants (Moore et al., 2003; Cohen, 2006). In addition to the higher population densities in the developing worlds, the economic growth and modern life style of urban inhabitants is causing serious pollution of soil and water ecosystems (Henderson, 2002; Qadir et al., 2010; Ferreira et al., 2018).

Urban soils are considered a fundamental ecological asset for cities and land-use planning (Bastida et al., 2007; Pavao-Zuckerman, 2008). These soils deserve more attention, than ever, due to substantial increase in interests of urban dwellers to use them for urban agriculture, gardening, and landscaping. Urban agriculture and home gardens are not only a quick source of fresh vegetables and fruits but also have social and environmental benefits (Kim et al., 2014; Al-Mayahi et al., 2019). Nonetheless, urban gardening should be practiced judiciously to protect soil and groundwater from many contaminants.

The sustainable management of soil and water resources is very critical in urban environment, particularly, in the developing countries (Pavao-Zuckerman, 2008; Qadir et al., 2010; Camotti et al., 2018). A very crucial issue in the sprawling urban dwellings is the handling and management of municipal wastes and sewage water (Grodzińska-Jurczak, 2001; Qadir et al., 2010; Mesjasz-Lech, 2014). Many of the urban citizens in the developing world have no access to proper disposal of municipal waste and sewerage systems which is resulting in severe pollution of their surroundings (McMichael, 2000; Qadir et al., 2010; Solgi et al., 2018). Besides, many urban residents deliberately or unintentionally aggravate soil and water pollution by the inappropriate management and handling of their waste materials. Globally, 20 Mha of land is irrigated with urban wastewater (Scott et al., 2004). Although, in many developing countries the use of untreated wastewater is banned for irrigation of crops, particularly those that are consumed raw, yet many farmers in these countries are applying untreated wastewater for irrigation in urban gardens with different justifications. Possibly, some people may know that wastewater could have many contaminants while others may not be aware of the fact that untreated wastewater is a good habitat of many human pathogens (Qadir et al., 2010; Raja et al., 2015). Nonetheless, numerous reports in literature reveal that the untreated municipal wastewater contains pathogenic microorganisms which if transfer to the surface and groundwater bodies could result in the outbreak of diseases (Burton et al., 1987; Donovan et al., 2008; Musyoki et al., 2013; Pandey et al., 2014; Rivera-Jaimes et al., 2018).

Typically, governments of most countries are considered responsible for finding out the most plausible strategies and development of legislations to keep the urban environment clean and sustainable, nevertheless, the role of residents is also very critical in keeping their urban environment healthier and cleaner. Thus, the knowledge and perception of the urban communities regarding the causes of environmental deterioration and their awareness about sustainable handling and management of valuable resources is imperative for healthy and clean environment. Hence, collaborative scientific and social studies are highly necessary to educate people about the environment and to increase the awareness regarding handling and management of soil and water resources that affects the sustainability of the urban environment. To that end, this study was conducted to find out the level of consciousness of people about handling of soil and wastewater and their awareness about the possible impacts of their farming, gardening, and municipal waste disposal activities on the surrounding soils and the contamination of soil and groundwater with the pathogenic bacteria.

## 2 Materials and methods

### 2.1 Ethics statement

This study was approved by The Research Council (TRC) of Oman with the Project No FRB/SQU/13/003. For the questionnaire part of this study, the urban residents consent was obtained then questionnaires were provided them. Permission was sought from individual home/garden owners for the collection of soil, groundwater and untreated water samples for pathogens analysis. All the guidelines for personal care were followed during sample collection and analysis of pathogenic bacteria.

### 2.2 Study area

**Fig. 1:**
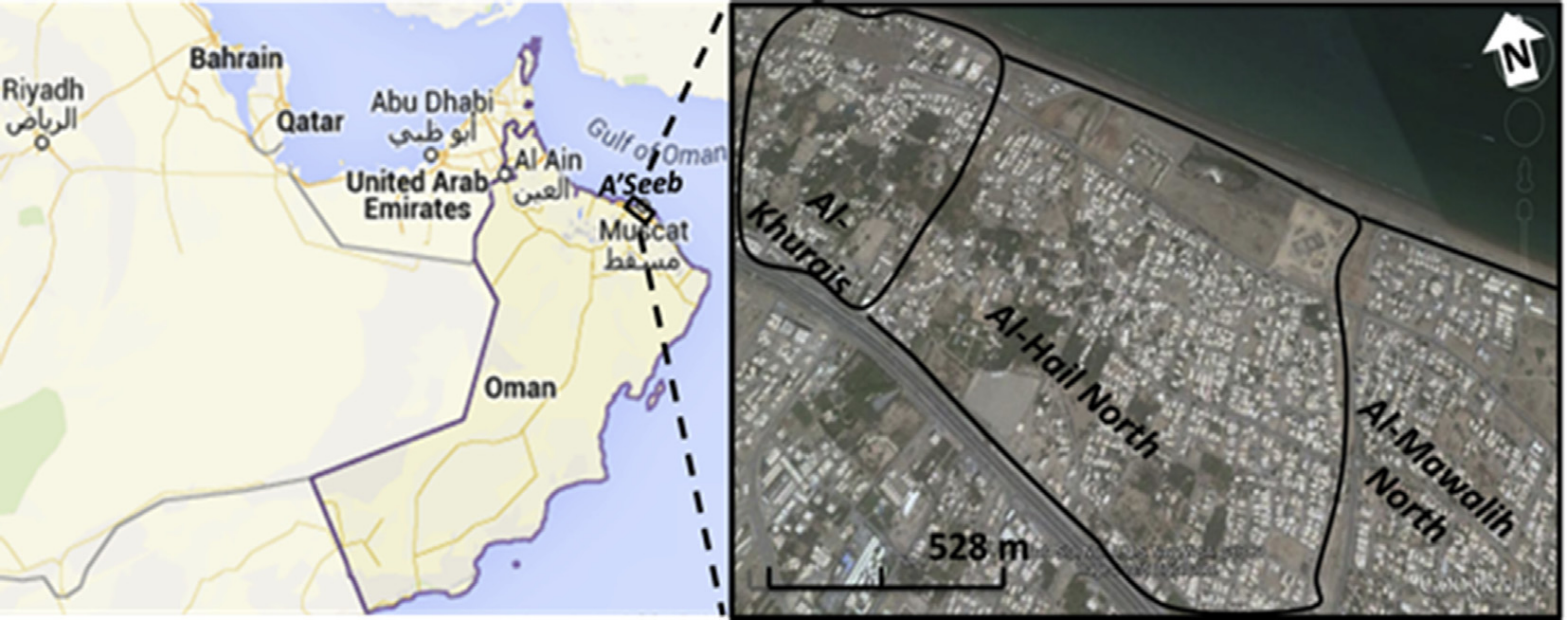
Google Earth^©^ map for the Sultanate of Oman and studied urban areas.

This study was carried out in the urban area of A'Seeb, one of the largest cities in the Sultanate of Oman. Sultanate of Oman is a rapidly urbanizing country where in 2017 the urban population was 78.5% of the total 4,636,000 population, and is expected to rise up to 86% in the year 2050 (World Bank, 2017). A'Seeb is located in the northwest part of Muscat Governorate (Fig. 1). It has an arid climate with an average annual rainfall of less than 100 mm and mean monthly air temperature ranging from 21 °C to 35 °C (winter–summer) with very hot extended summers, peaking up to 50 °C (Baawain and Al-Futaisi, 2014). A'Seeb was selected for this study because it had the highest percentage (34.1%) of housing units and households in the Muscat Governorate ( See supplementary material S1) (Ministry of National Economy, 2010). Moreover, this study was carried out on three major residential areas, namely Al-Khurais, Al-Hail North, and Al-Mawalih North, which were relatively old, urbanized and densely populated areas of the A'Seeb city.

### 2.3 Preliminary survey

A preliminary survey was conducted on these areas to explore the most common soil manipulating anthropogenic activities that may have an impact on the characteristics of the urban soils of A'Seeb. The most common system for sewerage water collection is underground septic tanks. Home owners had to request private operators to pump out sewage from the septic tanks at relatively high charges. Therefore, many residents got rid of the sewage water by various means. For example, many residents were irrigating their edible crops with untreated greywater or black water as part of home-gardening activities.

**Fig. 2:**
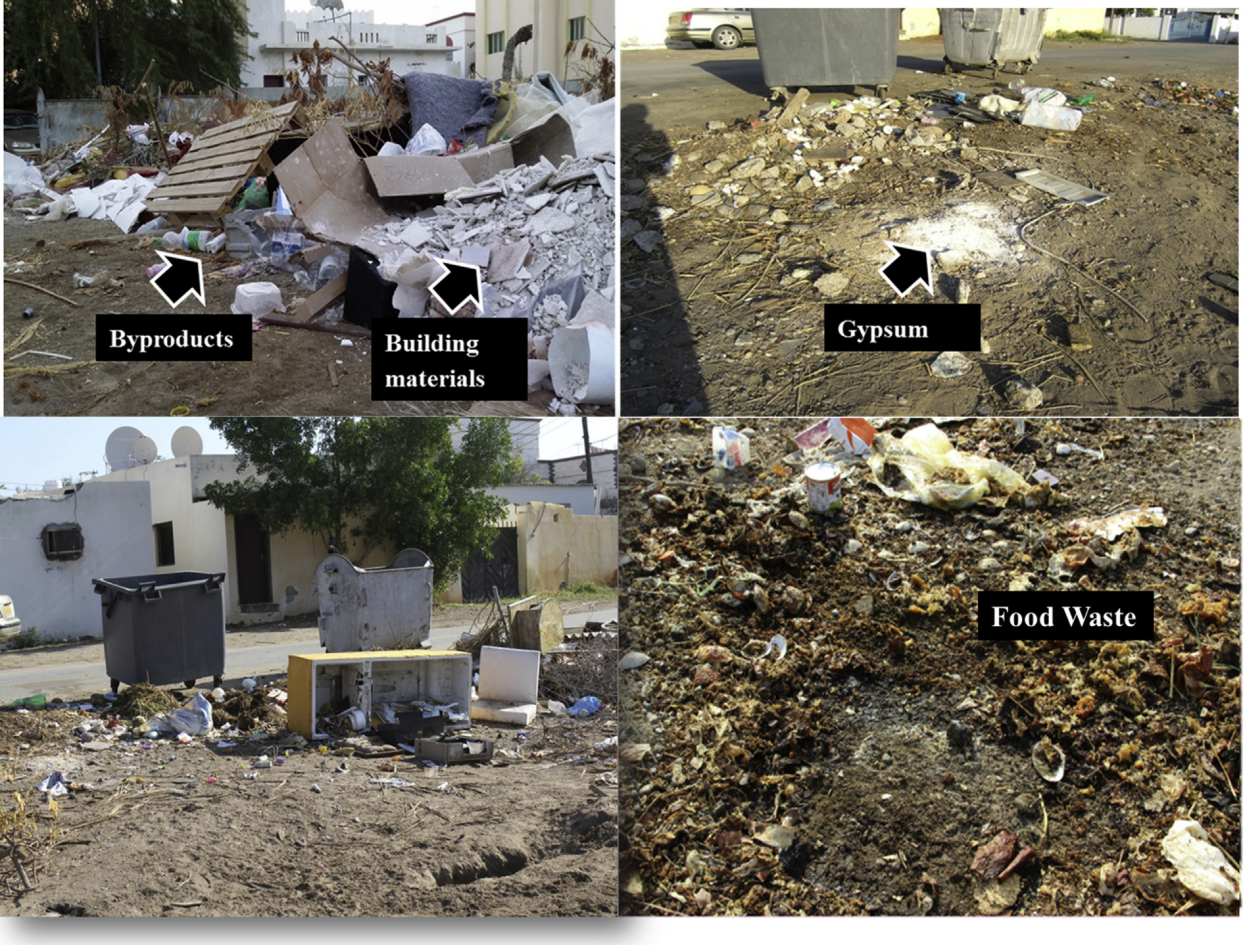
Food and other discarded materials on the garbage disposal sites.

In addition, the soils of the selected study area were exposed to household wastes as the residents were not properly dumping their garbage into the garbage disposal bins provided by the city municipality (Fig. 2). As the city was developing rapidly, there were many construction activities and significant amount of the construction byproducts and bitumen wastes etc. were also disposed on the soil.

### 2.4 Questionnaire

A questionnaire was prepared to discern knowledge and perception of the urban community residents about handling and management of municipal wastes and wastewater for urban gardening and possible soil contamination risks (See supplementary material S2). The questionnaire was designed based on guideline of De Vaus (2013) to obtained data about socio-economic characteristics of the respondents, knowledge about soil management activities; and the awareness about soil contamination due to throwing garbage materials outside the containers and irrigating with untreated greywater and black water. The design of the questionnaire was improved based on comments collected from the pilot questionnaire. Residents from 75 houses were randomly selected to fill out the questionnaire.

### 2.5 Statistical analysis

Categorical and binary response questions were analyzed using chi-square goodness of fit test and a one sample binomial test in R (version 3.2.1), respectively. P values <0.05 were considered significant. A linear regression model using Scheffe's multiple comparison test of differences between the means was employed in SAS software (SAS Institute, 2011; version 9.3) to assess the effects of disposal of municipal-waste and source of irrigation water (i.e. untreated black and greywater, and groundwater) on the chemical properties of the soil. The variation of chemical properties along the depth of soil profiles as a result of dumping garbage was also assessed. The interaction effect between soil properties and depth was dropped from the model because it was not significant (p < 0.05).

### 2.6 Soil and groundwater samples collection

A total number of 7 soil pedons were excavated in the study area where 5 of them were dug at the garbage site and 2 away from garbage site as a control. Soil profile morphology including artifacts or “anthropogenic particles” were described and classified according to USDA-NRCS Soil Survey methods (Schoeneberger, 2012; Howard and Orlicki, 2016). No pedons could be excavated on the home gardens sites due to private ownership. Soil samples were collected at an interval of 15 cm in each profile of the excavated pedons up to 120 cm. Additionally, top soil samples (0–30 cm) were collected from three home gardens; from a garden where untreated black water was used for irrigation, a second garden where untreated grey water was used for irrigation and a third garden where groundwater was used for irrigation. For each sampling site, four sub-samples were collected randomly at different locations within the gardens and on garbage disposal sites as well as respective controls. All collected soil samples were air dried, sieved (<2 mm) and analyzed for physico-chemical characteristics including soil texture, saturated hydraulic conductivity, pH, and salinity (EC_e_), following standard procedures (Dewis and Freitas, 1970; Gee and Bauder, 1986; Rhoades, 1996). Soil samples were also analyzed for water soluble heavy metals and other elements (i.e. As, Cd, Cu, Zn, Fe, Si, S, B, Pb, Na, Mg, and K) following standard methods using inductively coupled plasma mass spectrometry (ICP-MS)-mass (PerkinElmer). Additional samples were also collected for microbial analysis. A maximum of two sites were sampled each week and analyzed. The samples were collected aseptically at depth of 0–20 cm using a stainless steel spade and immediately stored in sterile plastic bags. The spade was washed with de-ionized water and sterilized with 70 % ethanol and wiped dry with paper towels after each use. About 300 g of soil was taken for each replication.

According to the questionnaire results, most gardeners were found to irrigate with untreated wastewater in Al-Khurais area, therefore, irrigation water samples were collected from four sites aseptically. Specifically, samples were collected from three wells and one septic tank. Well 1 was located in an area dense with houses (25 houses in a radial distance of 120 m) where many people applied untreated wastewater for irrigation. Well 2 was located 317 m away from Well 1 and had fewer houses surrounding it (4 houses in a radial distance of 120 m). Well 3 was located 313 m away from Well 1 and was inside an urban garden with very few house (5 houses in a radial distance of 120 m). In total, four samples were collected in each site. In order to make sure to get a reliable and representative groundwater sample in each well, the pump was opened to run for 30 minutes after which the water samples were collected.

### 2.7 Enumeration of pathogenic bacteria

All collected top soil samples were analyzed for the presence of total and fecal coliform, *E. Coli*, Salmonella, Shigella, and Staphylococci on the same day of sample collection using standard cultural methods (Eaton et al., 2005). Total coliforms and fecal coliforms were determined according the Most Probable Number (MPN) method using 15 Lauryl Tryptose Broth filled tubes (Woomer, 1994). First of all, in the presumptive test, 15 Lauryl Tryptose Broth tubes were inoculated with irrigation water samples or soil solutions (10 g soil suspended in 95 mL of sterilized distilled water). Then out of fifteen tubes, 10 mL of the sample was added in five tubes, 1 mL in other five tubes and 0.1 mL in the last five tubes. At that point, the fifteen tubes were incubated at 37 °C for total coliform and at 41.5 °C for fecal coliform, both for 24 ± 2 hours after which the gas production was determined. Tests were considered negative for no gas production in durhum tubes and positive when there was gas production in durhum tubes. The number of the positive tubes were compared to MPN Index (developed by the American Public Health Association) to calculate most probable number of coliforms. For confirmation, 0.1-mL from three of presumptive positive test (from each Total and Fecal Coliform) were inoculated into Brilliant Green Lactose Bile Broth (BGLBB) and incubated at 37 °C and 41.5 °C for total and fecal coliforms, respectively, for 24 ± 2 hours. Finally, the BGLBB was considered positive whenever gas was produced in durhum tubes.

For enumeration of *E. Coli*, Salmonella, Shigella and Staphylocooci, serial dilutions up to 10^7^ were made in Maximum Recovery Medium (MRD). Then the dilutions were transferred to respective selective medium for *E. Coli*, Salmonella, Shigella, and Staphylococci. For the enumeration of *E. Coli*, Tryptone Bile X-Glucuronide (TBX) was used and the plates were incubated at 37 °C for 24 ± 2 hours to determine the colony forming units (CFU) of *E. Coli* per g of soil which appeared as blue colonies. The counts of Salmonella and Shigella were determined by using Xylose-Lysine Deoxycholate (XLD [Becton Dickson]) agar and Bismuth Sulphite (BAS) Agar and plates were incubated at 37 °C for 24 ± 2 hours. Staphylococci were determined by taking a 0.1 mL of the serial dilution from each replicate and added into Baird Parker Agar (BPA) that were incubated at 37 °C for 24 ± 2 hours. Then, the CFU g^−1^ of the soil was determined by counting black colonies with halo zone.

### 2.8 Identification of pathogenic bacteria

From each selective medium, some colonies were selected on the basis of colony morphology and purified by streaking colonies on the respective selective medium for each microorganism. This process was performed 4–5 times to get a pure strain of each bacteria then the genomic DNA was extracted using MoBio DNA extraction kits (MoBio, Carlsbad, CA, USA) according to the manufactures’ protocol. The selected bacteria were identified using 16s rRNA gene sequencing. The PCR was performed as described by (Nadeem et al., 2012). The sequences obtained were compared with those found at the National Center for Biotechnology Information (NCBI) using BLAST search (https://blast.ncbi.nlm.nih.gov/Blast.cgi?PAGE_TYPE=BlastSearch). Then the 16s rRNA gene sequences were submitted to gene bank and their accession numbers were obtained.

## 3 Results

**Fig. 3:**
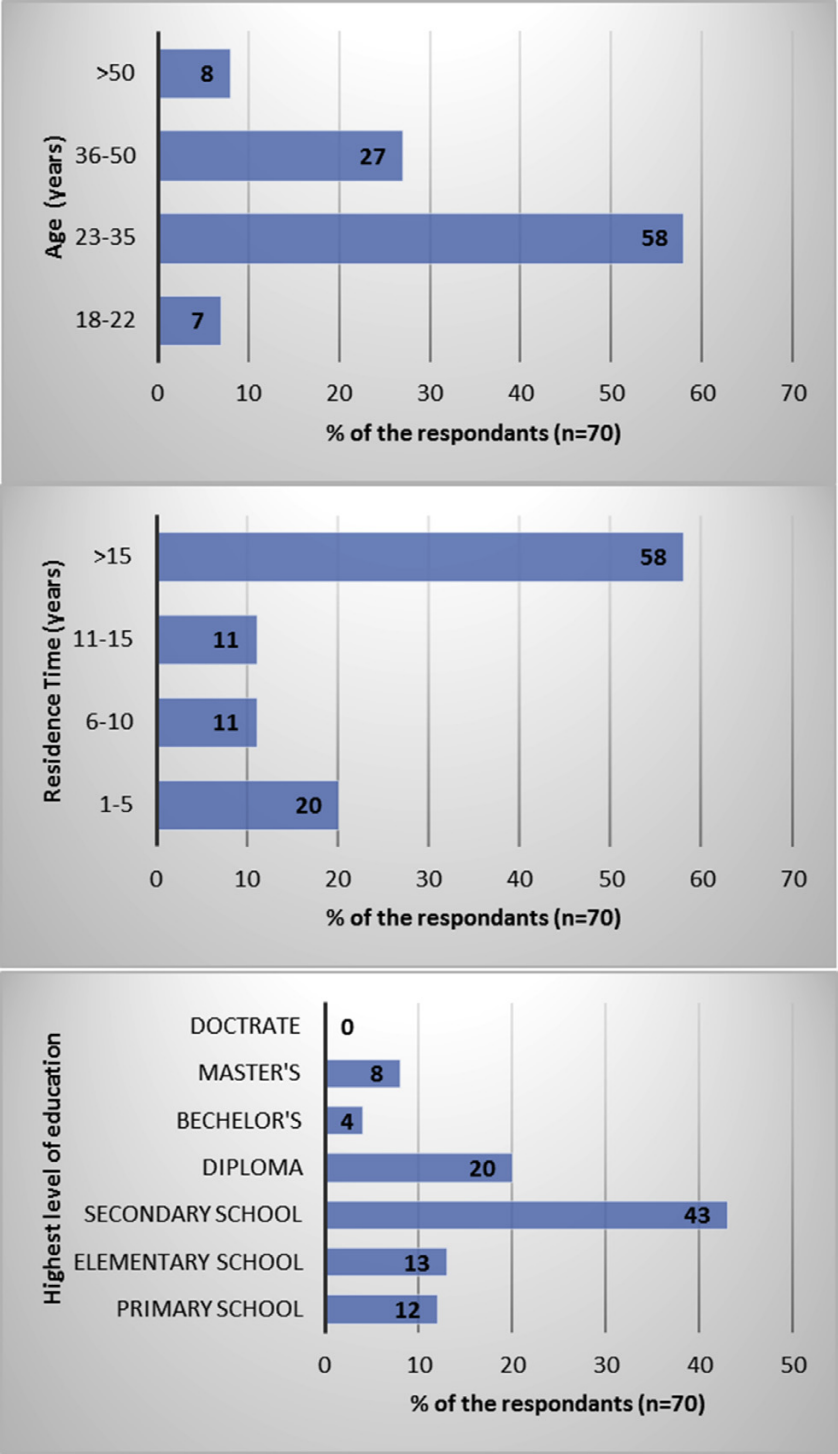
Demographic information of the respondents from the urban areas of A'Seeb, Oman.

People from about seventy-five residential houses responded to this studies' questionnaire. The demographic information of the residents of A'Seeb is summarized in Fig. 3. Most participants who filled the questionnaire were adults, 58% of the respondents were 22–35 years old, and 27% were 35–50 years old. Majority of respondents (58%) have been residing in their homes for the past 15 years. Many of the respondents (68%) had a basic level of education, with 43% having secondary school certificates. Only 20% of them had some diploma education, which was a relatively higher professional certificate than basic secondary school certificate. Very few respondents had a bachelor (4%) or master's degree (8%). Most of the respondents were male (96%) and almost all of them were practicing home gardening.

**Fig. 4:**
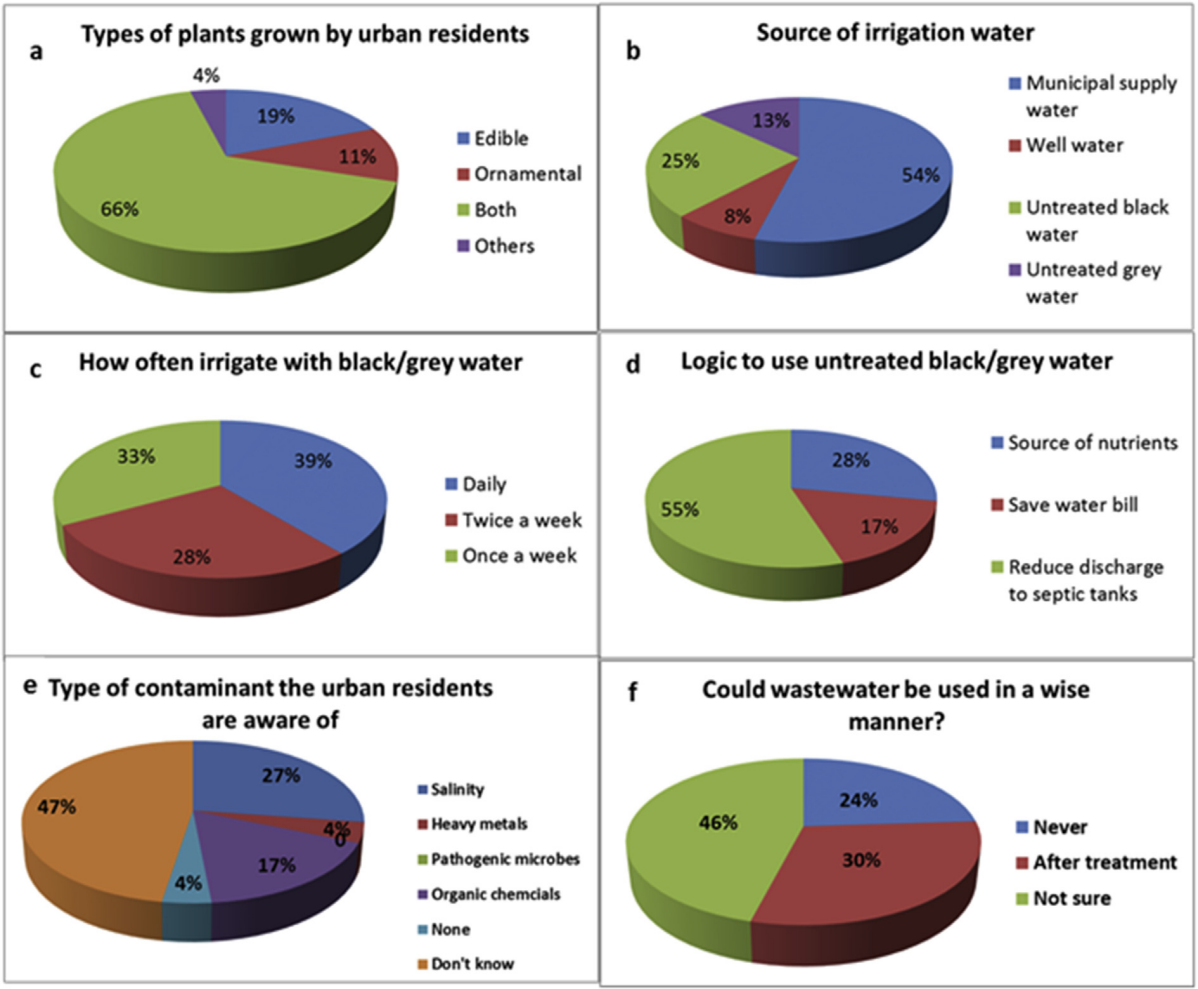
(a–f). Response of urban residents to some questions regarding use of untreated wastewater in their home gardens.

The response of residents to some close ended questions about home gardening is summarized in Table 1. Most of them showed interest in home gardening, wherein 42.3% were always interested and 51.3% were only sometimes and very few (6.4%) were never interested. Most of the people were cultivating both edible and ornamental plants (Fig. 4a) and the majority were growing for home consumption (p < 0.005) (Table 1). According to the survey data, most of the gardeners of the three residential areas were often applying farm yard manure to promote soil fertility while the others used chemical fertilizers. However, 18.7 % of the respondents were not using any type of fertilizers as they were relying on the natural fertility of the parent soil (Table 1).

**Table 1:** Response of the residents of the urban areas of A'Seeb to some close ended questions regarding home gardening (% of the respondents in various categories is given as well as the number of respondents (n)). P-values obtained using chi-square goodness of fit test for each question are shown.

Question	Always	Sometimes	Never	n	P-Value
Are you interested in home gardening?	42.3	51.3	6.4	78	1.855e-06
Do you consume the edible crops, fruits, vegetables etc. from home gardens?	67.6	27.9	4.4	68	8.92e-10
Do you apply fertilizers?	34.7	46.7	18.7	75	0.009069

Regarding the sources of water used for gardening (Fig. 4b), more than half of the surveyed households were applying municipal supplied water, but quite a big number were found to apply wastewater (38%), where in 25% were applying untreated black water (including all wastewater from toilets, bathrooms, kitchen and laundry) for irrigation and remaining 13% were applying untreated greywater (the wastewater which was mostly from kitchen and laundry). Only 8% of the respondents were applying well water (groundwater) for irrigation. Many of the residents were irrigating their plants daily (39%) or twice a week (28%), and the remaining respondents were applying irrigation once a week (Fig. 4c). When people who were using wastewater for irrigation (n = 27) were asked for the reason of this (Fig. 4d), a large percentage (55%) claimed that they did so "in order to reduce discharge to septic tanks". Residents usually had to pay the high prices to private operators for getting rid of wastewater from septic tanks. The other 17% of the respondents stated that they were applying wastewater in order to reduce bills of municipal supplied water as the residents had to pay for the amount of municipal water they consumed. Since, a decent amount of water was required to irrigate home gardens, many households used wastewater for irrigation to reduce their municipal water usage. The remaining 28% of the respondents used wastewater for irrigation because they believed that it was rich in nutrients and could help in improving plants growth and yield. Several examples of soil contaminants were presented to the participants to assess if they could identify soil contaminant they may had in their soil (Fig. 4e). Most of them (47%) were unaware of any soil contaminant. Others believed that the soil might contain some contaminants like, salinity (27%), organic chemicals such as oil, pesticides (17%) and heavy metals (4%). Nobody accepted that they had any pathogenic contamination in their soils and 4% of them were thinking their soil had no contamination at all. When residents were asked about the best and the wisest way to use untreated black and greywater, many of them (46%) were not confident about the best method, while other (30%) said it could be used safely after treatment. On the other hand, few (24%) of them were not in favor of using wastewater even after treatment (Fig. 4f). The responses of the urban residents to the close ended (yes or no) questions regarding handling and management of wastewater and garbage are presented in Table 2. In response to the question about soil testing ("Have your soils been tested?") nearly all of them (97.4%) replied with a no. However, the majority (72.4%) thought that soil testing was important for determining soil fertility, soil contamination, and soil sustainability. Other respondents had the idea that soil testing was not important and one of them commented “*why do I need to test my soils since my plants grow and produce well?! My soil is free of problems and away from contamination source like industries”*. Surprisingly, the response of most of the residents (79.5%) to the question whether they had ever heard of any regulations with regard to the direct use of untreated black or greywater was “No”. However, few of the residents (20.5%) replied that they had heard about that information either from their friends or from the employees of Ministry of Regional Municipalities and Water Resources (MRMWR).

**Table 2:** Response of the residents of the urban areas of A'Seeb to some close ended questions regarding handling and management of wastewater and garbage analyzed using one sample binomial test (% of the respondents, n and p values are given).

Question	Yes	No	n	P-value
Have you ever tested your soil?	2.6	97.4	76	2.2e-16
Do you think soil testing is important?	72.4	27.6	76	0.0001206
Have you ever heard about any regulation regarding use of untreated black or grey water?	20.5	79.5	73	1.691e-05
Have you or your family members ever felt that use of untreated black or grey water resulted in generating diseases like diarrhea, abdominal pain, and skin rashes?	34.4	65.5	61	0.02041
Have you ever seen that the people or municipality workers moved garbage containers from one place to another in the vicinity?	44.2	55.8	77	0.362
Have you heard about selective waste disposal system?	56.6	43.4	76	0.3019

Furthermore, when asked if they felt that using untreated black or greywater might had caused diseases like abdominal pain, diarrhea or skin rash for them or in their family, the majority (65.5%) replied that they never found any association between use of wastewater and diseases. Most of the respondents (88%) were aware of the fact that throwing garbage out of the container can cause soil contamination. When asked about the reason of doing so, majority (74%) attributed it to carelessness (See supplementary material S3). The site of garbage containers was not fixed at many locations as 44.2% of the respondents attested the displacement of garbage containers from one place to another in the same area (Table 2). The urban residents were also asked if they had some knowledge about selective disposal system and 56.6% replied “yes” to that question.

The questions and answers regarding the awareness of the residents of A'Seeb city about the contamination of the soil and water environment as a result of their activities are presented in Table 3. The answers were graded into strongly agree, agree, disagree, strongly disagree and do not know. Many of the residents had strongly agreed (40.3%) or agreed (35.1%) that soil could be contaminated with disease causing microorganisms if irrigated with black or greywater. Moreover, a good number of the residents believed that irrigation with black or greywater could even contaminate the ground water (40.3% strongly agree, 35.1% agree). About 75% of the surveyed residents had strongly agreed/agreed that the government should ban the use of untreated black or greywater. Also, almost all of them had agreed that the chemicals from the garbage materials could be transported or spread to the neighboring soil if thrown out of the containers. Furthermore, most of the residents had strongly agreed (32%) or agreed (31%) that throwing garbage onto the soil could lead to the propagation of disease causing microorganisms. When asked if they thought that the selective disposal system for the garbage could reduce the risk of pollution in urban environments, most of them had strongly agreed (60%) or agreed (29%). Keeping in view the handling and management of wastewater and soil by urban gardeners, soil samples were collected from gardens and garbage disposal sites and were studied for physical, chemical, morphological and quantification of selected pathogenic and fecal indicator bacteria.

**Table 3:** Response of the residents of the urban areas of A'Seeb to some questions regarding their awareness about the contamination of soil and groundwater as a result of their activities (% of the respondents, n and p values are given). The results were analyzed using chi square goodness of fit test.

Question	SA	A	D	SD	DK	n	P
Soil could get contaminated with disease-causing microorganisms if irrigated with untreated black or grey water?	40.3	35.1	5.2	2.6	16.9	77	3.867e-09
Irrigation with untreated black or grey water could contaminate groundwater?	40.3	35.1	5.2	2.6	16.9	76	1.48e-08
Should government ban the use of untreated black and grey water?	39.2	36.5	16.2	8.1	-	74	0.0001257
Throwing garbage onto the soil could lead to surface soil and ground water contamination	32	52	6.7	1.3	8	75	3.787e-14
Chemicals from the garbage materials could be transported to neighboring soil if thrown out of containers?	36.4	48.1	2.6	1.3	11.7	77	7.322e-14
Throwing garbage out of containers could increase the propagation of disease causing microorganism?	36.03	31.0	4.0	1.0	5.0	77	7.21e-15
Do you think selective disposal system for garbage could reduce risk of pollution in the urban environments?	60	29.3	4	4	2.7	75	2.2e-16

SA: Strongly agree; A: Agree; D: Disagree; SD: Strongly disagree, DK: Don't Know.

**Fig. 5:**
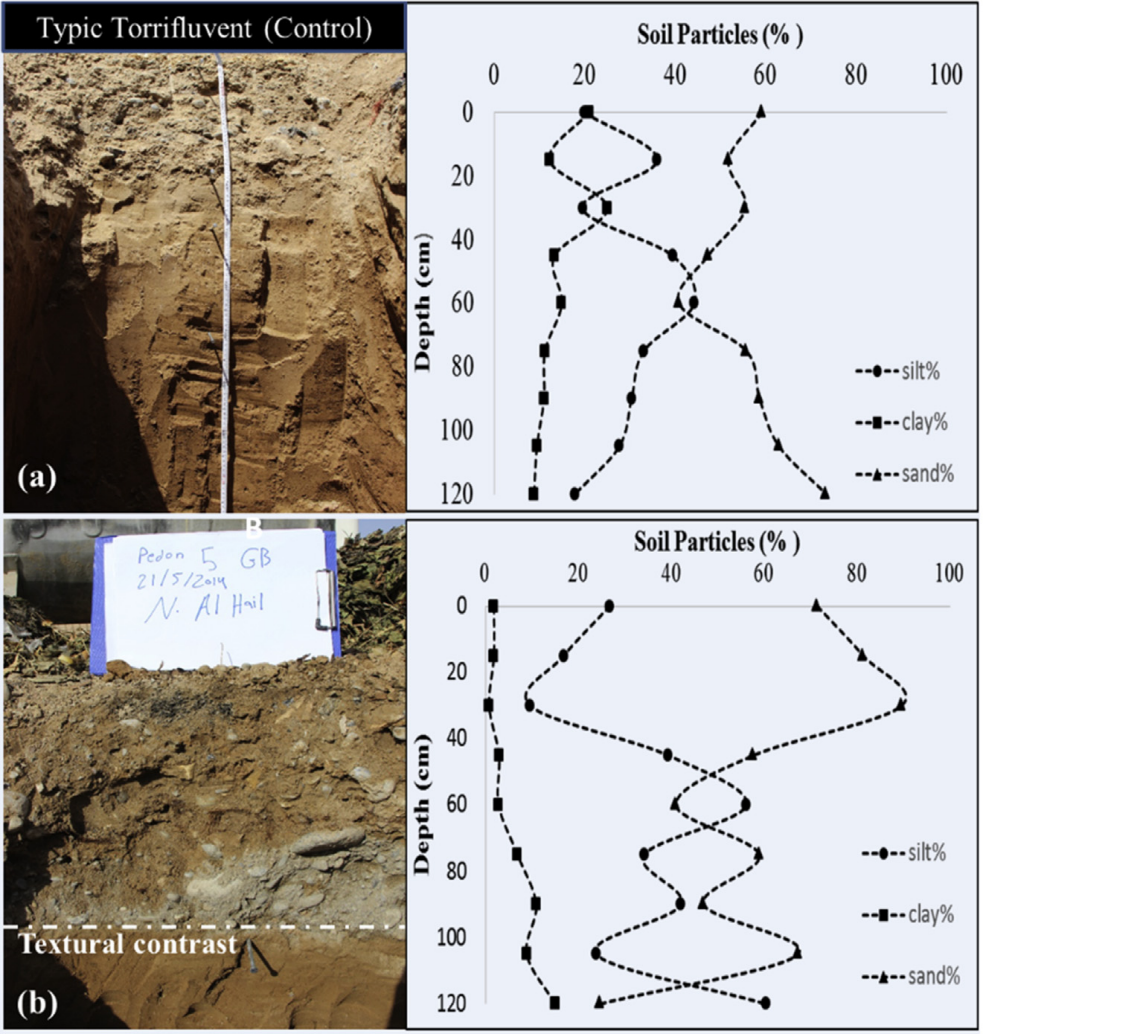
a,b. Particle size distribution with depth for soil profile of (a) the control compared to (b) garbage site.

**Fig. 6:**
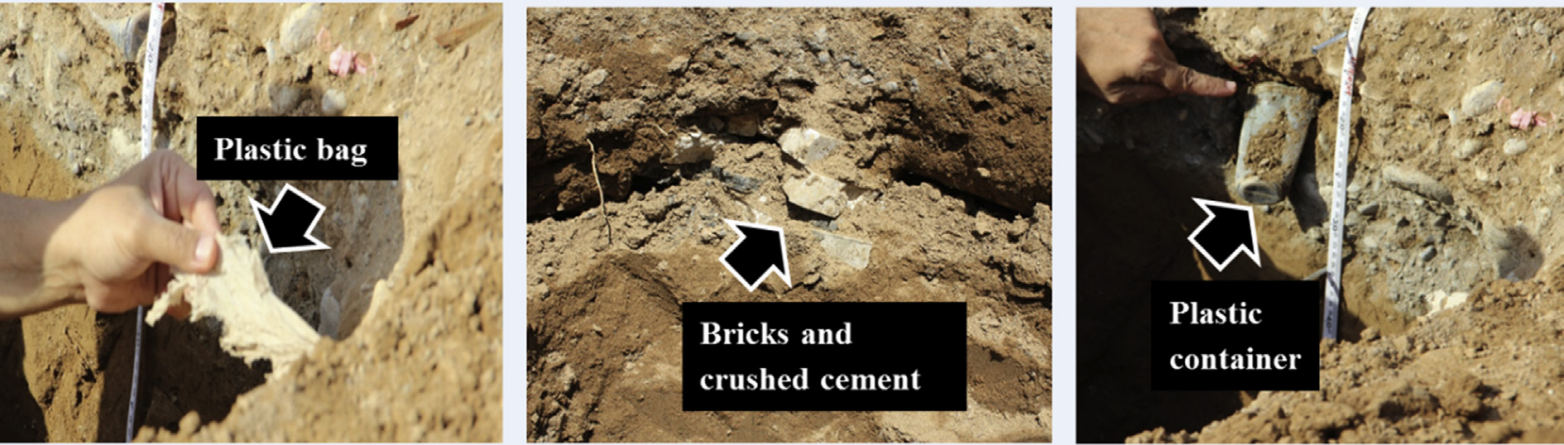
Artifact in the subsoil soil of garbage sites.

The soil types of the study area were dominated by Typic Torrifluvents. There were some few Typic Torriorthents and Typic Haplosalids soils on this study area (See supplementary material S4 for full profile descriptions of selected pedons). Figure 5a, b shows particle size distribution with depth of two selected profiles of the Torrifluvents at the garbage site and its respective control. The top 40 cm layer of the profile at the garbage site (Fig. 5b) had higher percentage of sand, 1.5x more, and less of silt and clay as compared to the control (Fig. 5a). Moreover, the measured saturated hydraulic conductivity of top horizons of profile at the garbage site was 507 cm^3^ day^−1^ whereas it was 11 cm^3^ day^−1^ for the control site. This postulates possibilities for transporting or infiltrating contaminant from the surface to deep soil layers or even groundwater especially during rain events at the garbage sites. Fig. 5b also shows an obvious textural contrast on top of the subsoil of the original Torrifluvents soil due to anthropogenic impact. In addition, profiles at the garbage sites had a presence of different type of artefacts e.g. bricks, plastic bags and containers, glass, rusted metals, car batteries, crashed cement, broken tiles, etc. at a depth of 0–50 cm (Fig. 6).

Estimated least square mean concentrations of selected chemical properties of soil profiles dug at the garbage disposal sites are shown in Table 4. The average value of soil pH and EC_e_ were significantly higher than the respective control only in profile 7 (Table 6). Among the elements, Cd, Cu and Fe were significantly higher than respective control in profiles 5, 6 and 7. However, the concentration of these elements did not exceed the EU (2002) and US EPA (2010) standard. Further, the statistical analysis showed no significant differences (p > 0.05) in the chemical properties along the depth of soil profiles (Table 6).

**Table 4:** Estimated least square mean concentrations and standard errors of selected chemical properties for soil profiles at the garbage sites.

Treatment	pH	ECe	Na	Mg	K	Ca	As	Cd	Cu	Zn	Fe	Si	S	B	Pb
P1	7.8 ± 0.15	0.51 ± 12.6	108.92 ± 31.8	43.4 ± 15.9	28.87 ± 14.4	75.37 ± 87.3	0.1639 ± 0.14	0.0144 ± 0.33	0.0001 ± 6.01E-6	0.0029 ± 0.006	0.0146 ± 0.005	0.7747 ± 0.25	41.5534 ± 8.84	0.1648 ± 0.059	0.0107 ± 0.036
P2	7.3 ± 0.15	4.46 ± 12.6	92.86 ± 31.8	82.34 ± 15.9	68.25 ± 14.4	443.54 ± 87.3	0.5422 ± 0.14	0.0178 ± 0.33	0.0001 ± 6.01E-6	0.0284 ± 0.006	0.0203 ± 0.005	1.90678 ± 0.25	50.1073 ± 8.84	0.2351 ± 0.059	0.1047 ± 0.036
P3	8.1 ± 0.15	0.43 ± 12.6	16.14 ± 31.8	6.07 ± 15.9	6.09 ± 14.4	9.38 ± 87.3	0.3994 ± 0.14	0.0158 ± 0.33	0.0001 ± 6.01E-6	0.007 ± 0.006	0.0141 ± 0.005	0.8483 ± 0.25	2.2985 ± 8.84	0.0945 ± 0.059	0.0003 ± 0.036
p4	8.1 ± 0.15	0.41 ± 12.6	3.68 ± 31.8	9.24 ± 15.9	N/A	46.11 ± 87.3	0.3347 ± 0.14	0.0128 ± 0.33	0.0001 ± 6.01E-6	0.0003 ± 0.006	0.019 ± 0.005	1.4584 ± 0.25	15.4724 ± 8.84	0.1792 ± 0.059	0.0003 ± 0.036
p5	7.4 ± 0.15	9.52 ± 12.6	N/A	76.43 ± 15.9	49.1 ± 14.4	211.69 ± 87.3	N/A	0.4139 ± 0.33	0.0001 ± 6.01E-6	0.0035 ± 0.006	0.0525 ± 0.005	N/A	N/A	0.2107 ± 0.059	0.0371 ± 0.036
p6	7.2 ± 0.15	13.87 ± 12.6	N/A	2.99 ± 15.9	0.46 ± 14.4	0.467 ± 87.3	N/A	5.173 ± 0.33	0.0001 ± 6.01E-6	0.0219 ± 0.006	0.0641 ± 0.005	N/A	N/A	0.2196 ± 0.059	0.032 ± 0.036
p7	6.8 ± 0.15	85.03 ± 12.6	N/A	3.44 ± 15.9	0.34 ± 14.4	0.35 ± 87.3	N/A	4.5625 ± 0.33	0.0001 ± 6.01E-6	0.0059 ± 0.006	0.0456 ± 0.005	N/A	N/A	0.2994 ± 0.059	0.0169 ± 0.036
*International Standards (**Environmental Data Management Software (ESdat.), 2019**)*															
EU	-	-	-	-	-	-	-	3	140	300	-	-	-	-	300
US EPA	-	-	-	-	-	-	-	3	50	200	-	-	-	-	300

P1 is control for P2 and P3 is control for the rest N/A: values are not available.

ECe is in dS m^−1^; Concentrations of elements are in mg kg^−1^. EU: European Union (EU 2002) US EPA: United States Environmental Protection Agency (2010)

Table 5 shows the estimated least square mean concentrations of selected chemical properties of garden soils irrigated with untreated wastewater and groundwater. The use of untreated wastewater increased soil EC_e_, concentrations of basic cations (i.e. Mg, and Ca), heavy metals (i.e. Cd, Cu) and other metals (i.e. S and B). Concentration of Mg, Ca and S were significantly higher in UBWS3 as compared to the control (Tables 5 and 6). Cd and Cu were the only heavy metals accumulated at a significant level only for UBWS2. It was also found that irrigating garden soils with groundwater resulted in significant increase in EC_e_, Na, Mg, Ca, Zn, Fe, Si and B (Tables 5 and 6). However, the concentration of heavy metasl in garden soils irrigated with untreated wastewater or groundwater were still below the EU and US EPA standards.

**Table 5:** Estimated least square mean concentrations and standard errors of selected chemical properties for soil sampled collected from gardens.

Treatment	pH	ECe	Na	Mg	K	Ca	As	Cd	Cu	Zn	Fe	Si	S	B	Pb
C1	8.6 ± 0.09	0.37 ± 0.39	0.67 ± 17.9	0.33 ± 9.1	4.38 ± 2.6	0.46 ± 30.1	0.1915 ± 0.23	0.0134 ± 0.002	0.0001 ± 0.00001	0.001 ± 0.005	0.0061 ± 0.006	0.3327 ± 0.28	0.93247 ± 11.17	0.02667 ± 0.014	0.0003 ± 0.017
GWS	8.5 ± 0.09	3.68 ± 0.39	190.9 ± 17.9	72.31 ± 9.1	20.38 ± 2.6	44.37 ± 30.1	0.2012 ± 0.23	0.0184 ± 0.002	0.0002 ± 0.00001	0.036 ± 0.005	0.0544 ± 0.006	2.87927 ± 0.28	43.52937 ± 11.17	0.2797 ± 0.014	0.0421 ± 0.017
UBWS 1	8.3 ± 0.09	1.13 ± 0.39	2.26 ± 17.9	1.51 ± 9.1	4.12 ± 2.6	10.15 ± 30.1	0.2345 ± 0.23	0.0138 ± 0.002	0.0001 ± 0.00001	0.0038 ± 0.005	0.0142 ± 0.006	1.00667 ± 0.28	0.01037 ± 11.17	0.0831 ± 0.014	0.0006 ± 0.017
C2	8.1 ± 0.09	0.43 ± 0.39	16.14 ± 17.9	6.07 ± 9.1	6.09 ± 2.6	9.38 ± 30.1	0.3994 ± 0.23	0.0158 ± 0.002	0.0001 ± 0.00001	0.007 ± 0.005	0.0141 ± 0.006	0.84837 ± 0.28	2.29857 ± 11.17	0.0945 ± 0.014	0.0003 ± 0.017
UBWS 2	8.4 ± 0.09	0.9 ± 0.39	52.57 ± 17.9	13.68 ± 9.1	7.64 ± 2.6	64.9 ± 30.1	0.7644 ± 0.23	0.0309 ± 0.002	0.0002 ± 0.00001	0.0006 ± 0.005	0.0304 ± 0.006	2.05057 ± 0.28	20.20877 ± 11.17	0.1171 ± 0.014	0.0003 ± 0.017
UBWS 3	8.3 ± 0.09	2.94 ± 0.39	80.25 ± 17.9	64.63 ± 9.1	15.06 ± 2.6	337.89 ± 30.1	0.4121 ± 0.23	0.0188 ± 0.002	0.0002 ± 0.00001	0.0119 ± 0.005	0.023 ± 0.006	1.2837 ± 0.28	125.62967 ± 11.17	0.1013 ± 0.014	0.0347 ± 0.017
UBWS 4	8.3 ± 0.09	0.94 ± 0.39	5.88 ± 17.9	2.91 ± 9.1	4.33 ± 2.6	19.38 ± 30.1	0.7677 ± 0.23	0.0161 ± 0.002	0.0001 ± 0.00001	0.0003 ± 0.005	0.0179 ± 0.006	1.43747 ± 0.28	1.87767 ± 11.17	0.0885 ± 0.014	0.0043 ± 0.017
*International Standards(**Environmental Data Management Software (ESdat.), 2019**)*															
EU	-	-	-	-	-	-	-	3	140	300	-	-	-	-	300
US EPA	-	-	-	-	-	-	-	3	50	200	-	-	-	-	300

C1 is control for GWS and UBWS1; C2 is control for the rest N/A values are not available.

ECe is in dS m^−1^; Concentrations of elements are in mg kg^−1^. EU: European Union (EU 2002) US EPA: United States Environmental Protection Agency (2010)

**Table 6:** P-value at 95% confidence interval of multiple comparison for treatments and their corresponding controls.

	pH	ECe	Na	Mg	K	Ca	As	Cd	Cu	Zn	Fe	Si	S	B	Pb
***Profiles at the garbage site***															
p1 vs p2	0.618	1.000	0.987	0.799	0.6042	0.264	0.380	1.000	0.767	0.211	0.993	0.098	0.921	0.992	0.743
p3 vs p4	1.000	1.000	0.993	1.000	N/A	1.000	0.99	1.000	0.907	0.992	0.996	0.467	0.777	0.979	1.000
p3 vs p5	0.290	0.999	N/A	0.224	0.519	0.833	N/A	0.992	0.002	0.999	0.007	N/A	N/A	0.912	0.996
p3 vs p6	0.075	0.995	N/A	1.000	0.999	1.000	N/A	<0.0001	0.029	0.752	0.000	N/A	N/A	0.882	0.998
p3 vs p7	0.007	0.024	N/A	1.000	0.999	1.000	N/A	<0.0001	0.001		0.032	N/A	N/A	0.466	1.000
Overall P-Value for variation with depth	0.833	0.307	0.179	0.294	0.562	0.520	0.156	0.1091	0.308	0.825	0.308	0.562	0.149	0.095	0.495
***Gardens***															
Control 1 vs UBWS 1	0.7972	0.9176	1.000	1.000	1.000	1.000	1.000	1.000	0.999	0.999	0.988	0.821	1.000	0.332	1.000
Control 1 vs GWS	0.9961	0.0027	0.0003	0.005	0.0341	0.9785	1.000	0.7536	0.9532	0.0064	0.0069	0.0017	0.3565	<0.0001	0.8013
Control 2 vs UBWS 2	0.345	0.9916	0.9003	0.9989	0.9998	0.9351	0.9661	0.0056	0.002	0.980	0.760	0.253	0.966	0.979	1.000
Control 2 vs UBWS 3	0.887	0.0255	0.4235	0.0255	0.4594	0.0002	1.000	0.9682	0.332	0.995	0.981	0.973	0.0002	1.000	0.906
Control 2 vs UBWS 4	0.7972	0.9867	0.9999	1.000	0.9997	1.000	0.9646	1.000	0.997	0.976	0.999	0.894	1.000	1.000	1.000

The results revealed that garden soils irrigated with untreated black water were contaminated with a large number of pathogenic bacteria, including *E. Coli*, Staphylococcus, Salmonella, Shigella, and fecal coliforms (Table 7). On the contrary, no pathogenic bacteria (*E. Coli*, Staphylococcus, Salmonella, Shigella) and only some fecal indicator bacteria were detected in the control soils which were sampled only 2–3 m away from the untreated black water irrigated gardens. However, in soils irrigated with greywater, only total and fecal coliform were found, nonetheless, *E. Coli*, Staphylococcus, Salmonella and Shigella were not detected on the selective media. Likewise, there were only some coliform present in the control and no other pathogenic groups were seen. Surprisingly, soils irrigated with groundwater were also found to be contaminated with some total and fecal coliforms, though, their number were less than 100 CFU g^-1^ of soil. In the same way, no pathogenic bacteria were observed in the soil 2–3 m away from the well irrigated soil. The soils on the garbage disposal sites were also found to be contaminated with many pathogenic bacteria comprising of *E. Coli*, Staphylococcus, Salmonella, Shigella, total and fecal coliforms, however, no pathogenic bacteria were found in the control soils that were 3–4 m away from the garbage disposal sites.

**Table 7:** Pathogenic bacteria (CFU g-^1^ soil) in soils irrigated with different water sources (n = 4) and exposed to garbage (n = 20).

Soils irrigated with								
Soil	Untreated sewage water	Control	Grey water	Control	Well water	Control	Garbage soil	Control
E.Coli	2.6 × 10^5^ ± 9 × 10^3^^a^	0	0	0	0	0	5.0 × 10^3^ ± 3 × 10^2^	0
Staphylococcus	1.7 × 10^4^ ±1 × 10^3^	0	0	0	0	0	3.3 × 10^3^± 5 × 10^2^	0
Salmonella/Shigella	1.5 × 10^4^ ± 2 × 10^2^	0	0	0	0	0	1.2 × 10^3^ ± 1 × 10^2^	0
Total Coliform	>2400	54 ± 21	815 ± 49	71 ± 21	74 ± 18	22 ± 16	>2400	<2
Fecal Coliform	>2400	26 ± 19	801 ± 83	17 ± 10	49 ± 2	20 ± 13	>2400	<2

a Standard error.

The black water was also analyzed for the presence of pathogenic bacteria and high *E. Coli* count was detected in it. Similarly, a substantial density of total and fecal coliforms (>2400 CFU mL^-1^) were found (Table 8). However, Salmonella/Shigella were less than 100 CFU mL^-1^. For soils on sites irrigated with groundwater and contaminated with some coliforms, water sample were collected from wells nearby the gardens to test the presence of pathogenic bacteria. Well 1 which was close to Al-Khurais residential units, and where many people were using black water for irrigation was found to be contaminated with some staphylococcus, Salmonella/Shigella and had a high count of *E. Coli*. The total and fecal coliforms in well 1 were >2400 CFU mL^-1^. Well 2 was located at the place where the number of houses were comparatively less than the location of Well 1. The *E. Coli* count and coliform (total and fecal) were less than Well 1, however, the salmonella/Shigella counts were higher. Well 3, that was located away from residential units (inside an urban garden), was found to contain even less number of total and fecal coliforms (167 and 9 CFU mL^-1^, respectively). Nonetheless, the counts of Salmonella in Well 3 were higher compared to the other two wells (Table 8).

**Table 8:** Pathogenic bacteria (CFU mL^−1^ ± standard error) in irrigation water from different water sources in four different locations.

Water sample	E.Coli	Staphylococcus	Salmonella/Shigella	Total Coliform	Fecal Coliform
Raw sewage	2019 ± 912	0	98 ± 0.8	>2400	>2400
Well 1	1089 ± 157	13 ± 0.5	50 ± 1.0	>2400	>2400
Well 2	750 ± 60	0	95 ± 3.0	1660 ± 89	826 ± 75
Well 3	0	0	103 ± 6.1	167 ± 3.6	9 ± 3.0

The colonies on different selective media were selected randomly, purified and the individual strains of the bacteria were identified by 16S rRNA gene sequence analysis.

The data revealed that out of 16 identified isolates from the urban areas of A'Seeb, all isolates were well known human pathogens and 10 isolates belong to risk group 2 (Table 9). The dominant bacterial species were *Klebsiella spp.* followed by *E. Coli, Shigella spp.* and *Enterobacter spp.* All the bacterial isolates from black water irrigated soil were human pathogens and 4 out of 5 isolates from the groundwater irrigated soil were human pathogens and one isolate *Eb. aerogenes,* which is an opportunistic pathogen. However, from garbage disposal sites, three isolates were found to be pathogenic and one isolate *Eb. cloacae,* is an opportunistic pathogen (see Table 10).

**Table 9:** Identification of pathogenic bacteria isolated from urban soils of A'Seeb Oman.

Source	GPS location	Bacterial sp.	GBA#	Disease caused/risk group (Ref.)
UBWS	23°39′15.00″ 58°13′10.00″	*K. granulomatis*	KP209245	Genital ulcer (Mackay et al. 2006)
UBWS	23°39′15.00″ 58°13′10.00″	*Ec. faecalis*	KP209246	G2 (Canada 2009)
UBWS	23°39′15.45″ 58°13′10.87″	*K. oxytoca*	KP209247	Opportunistic pathogen (Lowe et al., 2012)
UBWS	23°39′15.45″ 58°13′10.87″	*Klebsiella sp.*	KP209248	G2
UBWS	23°39′15.70″ 58°13′9.19″	*Shigella sp.*	KP209252	G2
UBWS	23°39′15.70″ 58°13′9.19″	*E. coli*	KP209253	G2
GWS	23°37′56.70″ 58°14′35.60″	*K. pneumoniae*	KP209256	G2
GWS	23°39′14.60″ 58°13′11.40″	*K. pneumoniae*	KP209258	G2
GWS	23°39′17.49″ 58°13′8.60″	*E. coli*	KP209259	G2
GWS	23°38′10.14″ 58°14′3.39″	*Eb. aerogenes*	KP209261	Opportunistic pathogen (Thiolas et al. 2005)
GWS	23°38′10.14″ 58°14′3.39″	*K. pneumoniae*	KP209281	G2
GS	23°38′6.79″ 58°14′8.32″	*Staph. aureus*	KP209249	Skin infection, food poisoning (Lowy, 1998)
GS	23°38′12.90″ 58°14′0.30″	*S. sonnei*	KP209260	G2
GS	23°38′12.90″ 58°14′0.30″	*Kl. georgiana*	KP209282	Opportunistic pathogen (Carter and Evans, 2005, Sarria et al., 2001)
GS	23°38′6.20″ 58°14′20.90″	*Eb.cloacae*	KP209283	Bacteremia, UTI, CNS infection (Harbarth et al. 1999)
GS	23°37′47.00″ 58°15′8.50″	*E. coli*	KP209284	G2

GBA#, Gene bank accession number; (Ref), References; UBWS, Untreated black water irrigated garden soil; GWS, ground water irrigated garden soil; GS, Soil incorporated with garbage; *K, Klebsiella; S, Shigella, Kl. Kluyvera, E, Escherichia; Ec., Enterococcus; Eb., Enterobacter.*

**Table 10:** Identification of pathogenic bacteria isolated from different sources of irrigation waters in the urban areas of A'Seeb Oman.

Source	GPS location	Bacterial sp.	GBA#	Risk group/Disease caused (Ref.)
UBW	23°39′15.00″ 58°13′10.00″	*K. pneumoniae*	KP209250	G2 (Canada 2009)
UBW	23°39′15.00″ 58°13′10.00″	*Shigella sp.*	KP209254	G2
UBW	23°39′15.45″ 58°13′10.87″	*Eb. cancerogenus*	KP209255	Opportunistic pathogen (Wei et al. 2013)
UBW	23°39′15.45″ 58°13′10.87″	*K. pneumoniae*	KP209251	G2
UBW	23°39′15.70″ 58°13′9.19″	*E. coli*	KP209257	G2
UBW	23°39′15.70″ 58°13′9.19″	*A. caviae*	KP209264	Gastroenteritis (Altwegg 1985)
GW 1	23°39′17.1″ 58°13′08.7″	*K. pneumoniae*	KP209262	G2
GW 1	23°39′17.1″ 58°13′08.7″	*K. pneumoniae*	KP209263	G2
GW 1	23°39′17.1″ 58°13′08.7″	*Pseudomonas sp.*	KP209265	
GW 1	23°39′17.1″ 58°13′08.7″	*Eb. aerogenes*	KP209266	Nosocomial Pathogen (Thiolas et al. 2005)
GW 1	23°39′17.1″ 58°13′08.7″	*E. coli*	KP209267	G2
GW 1	23°39′17.1″ 58°13′08.7″	*Shigella sp.*	KP209268	G2
GW 2	23°39′07.9″ 58°13′13.7″	*Ec. faecalis*	KP209269	G2
GW 2	23°39′07.9″ 58°13′13.7″	*S. sonnei*	KP209270	G2
GW 2	23°39′07.9″ 58°13′13.7″	*Sw. decolorationis*	KP209271	
GW 2	23°39′07.9″ 58°13′13.7″	*K. milletis*	KP209272	
GW 2	23°39′07.9″ 58°13′13.7″	*K. pneumoniae*	KP209273	G2
GW 2	23°39′07.9″ 58°13′13.7″	*K. pneumoniae*	KP209274	G2
GW 3	23°39′10.2″ 58°13′16.7″	*S. flexneri*	KP209275	G2
GW 3	23°39′10.2″ 58°13′16.7″	*K. oxytoca*	KP209276	Opportunistic pathogen (Lowe et al., 2012)
GW 3	23°39′10.2″ 58°13′16.7″	*Shewanella sp.*	KP209277	
GW 3	23°39′10.2″ 58°13′16.7""	*P. stutzeri*	KP209278	
GW 3	23°39′10.2″ 58°13′16.7″	*Ac. parvus*	KP209279	
GW 3	23°39′10.2″ 58°13′16.7″	*Eb. ludwigii*	KP209280	

GBA#, Gene bank accession number; (Ref.), References; UBW, Untreated Black Water; GW, Groundwater well; *K*, Klebsiella; S, Shigella, E, Escherichia; Ec. Enterococcus; Eb, Enterobacter; A, Aeromonas; Sw, Shewanella; Ac, Acinetobacter.

Similarly, many isolates purified from untreated wastewater and groundwater were found to harbor pathogens (17/24 isolates) and 13 of them belong to risk group 2 (Table 9). The dominant bacterial species in the identified isolates was Klebsiella (8/24 isolates) followed by Shigella (4/24 isolates). All of the isolates from wastewater were human pathogens. Similarly, most of the isolates from Well 1 and Well 2 were pathogens (5 and 4, respectively, out of 6 isolates). However, only 2 isolates from Well 3 were found to contain pathogens or opportunistic pathogens.

## 4 Discussion

The soils of the urban areas are nowadays more exploited due to increasing human population, economic development and higher interests of urban dwellers in urban gardening. Urban residents could play vital role in keeping soils healthy and sustainable. Therefore, in this study, the knowledge of urban residents regarding urban gardening, soil handling and management of municipal wastes were assessed using a preliminary survey and a questionnaire. It was evident from the results of our study that urban residents were very much interested in urban gardening, specifically of edible crops and ornamental plants. Nevertheless, the way urban residents handle the soil was not acceptable and posed a potential health hazard. While most of the urban growers knew that soil testing was important, none of them had ever tested their soil for any nutrient or soil contamination. Many urban residents were unaware about any regulations or legislations regarding the use of untreated wastewater. Therefore, a good number of them were using untreated wastewater for irrigating their plants (Fig. 4b) even though they agreed that it might had contaminated the soil and groundwater with various contaminants including pathogenic microorganisms (Table 3). Amazingly, they believed that no pathogenic microbes were present in their soils (Fig. 4e). We postulate the reason behind this behavior to be driven by the high cost of irrigating with fresh water as well as disposal of sewage water (Fig. 4d). These facts have also been revealed in newspapers (Al-Shaibany, 2013, 2014) which showed that urban residents found it justified to get rid of wastewater from septic tanks by unconventional means instead of paying charges for disposing of it. Some residents said they had to pay up to 250 US$ per month for emptying their septic tanks which put a huge burden on their budget. Therefore, the general attitude was that of saving at the expense of environment with little to no concern about the resulting pollution. Kim et al. (2014) have also reported that the urban gardeners have little concern about the soil contamination, but in their study they have paid attention to all contaminants including heavy metals, organic chemicals, other chemicals, and biological hazards. In this study, we reported changes in the chemical properties of urban soils and in the quality of groundwater. Significant accumulations of basic cations (i.e. Na, Mg and Ca), some heavy metals (i.e. Cd, Cu, Zn and Fe) as well as Si, S and B were recorded in our study. These findings coincided with the results reported by Kiziloglu et al. (2007), Qishlaqi et al. (2008), Aydin et al. (2015), Abegunrin et al. (2016), and Rattan et al. (2005). Although some of the sites were significantly contaminated with heavy metals, concentrations were still far below the EU and US EPA standards. Similar findings of low concentrations of heavy metals and trace elements in the Omani cultivated soils were reported by Al-Busaidi et al. (2015).

The urban dwellers had also polluted the soil directly by throwing the garbage on to the soil out of sheer carelessness. According to their reports, the garbage bins were translocated from one place to another in the same locality which in turn increased the spread of contamination. Moreover, many artifacts (e.g. bricks, plastic bags and containers, glass, rusted metals, car batteries, crashed cement, broken tiles, etc.) from garbage material could get buried to deeper depths by time rendering the soils unsuitable for any agricultural or gardening usage. This premise was supported from the observation while digging pedons at the places of garbage disposal, where many artifacts, including tiles, papers, plastics, bottles, cans, metals etc. were found in the subsoil soils too (Fig. 6). We have also found that the top soil horizons of some of the profiles at the garbage site had distinct changes in the soil texture dominated by sands with high saturated hydraulic conductivity, pH, EC_e_, and concentrations of Cd, Cu, and Fe (Table 4). Other studies have also documented that frequent dumping of municipal waste into the soil increased the total porosity, hydraulic conductivity, cation exchange capacity, and heavy metals (Anikwe and Nwobodo, 2002; Taylor and Allen, 2006).

When soils were tested for the quantification of fecal contamination indicator bacteria, many of well-known pathogenic groups including *E. Coli*, Staphylococci, Slamonella/Shigella and total and fecal coliform were detected, particularly, in soil irrigated with black water and where garbage was disposed. The number of Colony Forming Units (CFU) per gram of soils were much higher in the contaminated soils compared to the controls. It is most likely that if people get in contact with these soils or accidentally consume its particles, particularly the children who often play in their gardens and could ingest some soil (Kim et al., 2014), they could suffer from disease like diarrhea, pneumonia, stomach cramps, shigellosis, urinary tract infections etc. It is a well-known fact that the sewage water contains high amounts of many human pathogens (Jourda et al., 2006; Musyoki et al., 2013; Pandey et al., 2014). In many developing countries, people suffer from many diseases where untreated wastewater is used for irrigation (Qadir et al., 2010; Raja et al., 2015). Therefore, continues application of untreated wastewater could provide very suitable niches for the proliferation of pathogenic bacteria. It is evident from the data (Table 7) that black water irrigated soils and garbage disposal places contained more viable pathogenic bacteria as compared with the control soils which were only 2–3 meters away. Apparently, many of the bacterial species could die naturally (Burton et al., 1987; Epstein, 2002; Jiménez, 2006; Hentati et al., 2014), particularly, when exposed to high temperature which is very common in Oman; top soil temperatures in summer may reach up to 60 °C. Even then we were able to detect a large number of bacteria in the soil which could be attributed to the fresh load of viable microbes with their food in every irrigation with untreated black water that farmers applying every week. Many studies reported that the application of wastewater results in the accumulation of many pathogenic microorganisms including bacteria (Epstein, 2002; Jiménez, 2006; Hentati et al., 2014).

Surprisingly, some coliforms were found in groundwater irrigated soil, although their counts were relatively less. Therefore, we had collected groundwater samples in order to test the presence of pathogenic bacteria. Water samples from wells close to the places where untreated wastewater was used had high coliform and fecal coliform which deemed them unsuitable for irrigation (Blumenthal et al., 2000). Presumably, the groundwater might have been contaminated with pathogens due to irrigation of plants with untreated black water which might have moved to groundwater due to sandy soil texture and the shallow water table. Another reason could be the leakage of septic tanks (Al-Bahry et al., 2014; Baawain and Al-Futaisi, 2014) which also allows seepage of pathogens containing wastewater into the soil and eventually reaches groundwater. Also, to avoid cost of emptying septic tank, some urban dwellers deliberately make holes in septic tanks to delay their filling, which contaminates underground water and is hard to detect unless regular checks are performed by the government (Nebel and von Richthofen, 2016). In Wells 2 and 3, the coliform counts were relatively less compared with Well 1 (Table 8). This might be due to the fact that Wells 2 and 3 were having fewer houses. The coliform count was the least in Well 3 which might be due to the fact that this was located inside the garden and the gardener was irrigating with the municipal supply water. Contamination of groundwater with fecal indicator pathogens is common urban areas of developing countries where untreated wastewater is used for irrigation (Qadir et al., 2010; Raja et al., 2015). The spread of related infectious diseases is also very common in these countries. Groundwater contamination with pathogenic bacteria and diseases outbreaks have also been reported in some developed countries like USA, Canada, and France (Gallay et al., 2006; Hynds et al., 2014; Wallender et al., 2014), though, the situation is worst in the developing countries (Qadir et al., 2010; Raja et al., 2015).

Some of the typical bacterial colonies were purified and identified from each selective medium. Most of the identified isolates were human pathogen of risk group 2 (Government of Canada, 2014), including *E. Coli*, *K. pneumonia,* and *Shigella sp.* or other opportunistic pathogens. The most dominant bacterial species found were *K. pneumonia* and *Shigella sp.* It is most likely that the occurrence of many diseases including diarrhea, pneumonia, liver abscesses, stomach cramps, shigellosis, urinary tract infections etc. could increase in residents particularly children, in the urban areas. This premises is supported from the facts that these two bacterial species are found to be the emerging pathogens in Oman (Scrimgeour et al., 1999; Prakash, 2008; Basu, 2009; Ansari et al., 2012). Basu (2009) reported that during 2002–2007 two out of fifteen cases with liver abscesses were due to *K. pneumonia.* Similarly, *Shigella spp.* are found to be important causative agent of gastroenteritis, diarrhea and shigellosis in many clinical reports from Oman (Scrimgeour et al., 1999; Prakash, 2008). Moreover, it is documented in several reports that resistance of these human pathogens to many antibiotic is increasing in Oman (Prakash, 2008; Al-Bahry et al., 2014). The presence of multi-antibiotic resistant pathogens could be therefore even more dangerous as they could not only increase the spread of diseases but also could transfer antibiotic resistance to other organisms, including animals and urban residents themselves. This premise could pose dangers on the health situation in Oman and other developing countries where untreated wastewater is used for irrigation of crops.

## 5 Conclusions

Unfortunately, the majority of surveyed residents of A'Seeb area of Oman were not paying attention to the negative consequences of their activities on soil and environment. Most of them knew that using untreated wastewater and careless disposal of garbage could cause soil and groundwater contamination. Yet, they were applying untreated wastewater for irrigation and throwing garbage on the soil. Such practices appeared to initiate soil contamination of the A'Seeb urban soils by heavy metals, salts, and anthropogenic artifacts. Analysis, of soil samples clearly revealed that their activities had affected physical and chemical properties of these soils. Moreover, these urban soils were found to be contaminated with E. Coli, staphylococcus, salmonella and shigella, many of which had been reported as human pathogens.

The chances of many infectious disease including diarrhea, shigellosis, cholera, etc. and antibiotic resistance in human beings could increase if no stern measures are taken against the behavior of urban residents. Hence, rigorous efforts are needed to make urban residence mindful about their soil and water environments. In this study we used cultural techniques to determine the viable bacterial counts. Future studies should be done using high throughput genotyping techniques, including pyro sequencing to identify all possible types of pathogens including viruses in urban soils and groundwater. More socio-environmental studies are needed to further correlate the behavior of urban residents against pollution and spread of diseases. Finally, the results of this research set a foundation for future studies on urban soils and associated residence behaviors and practices in Oman and the neighboring Gulf countries.

## Declarations

### Author contribution statement

Said Al-Ismaily: Conceived and designed the experiments; Analyzed and interpreted the data; Contributed reagents, materials, analysis tools or data; Wrote the paper.

Baby Shaharoona: Analyzed and interpreted the data; Contributed reagents, materials, analysis tools or data; Wrote the paper.

Ahmed Al-Mayahi: Performed the experiments; Analyzed and interpreted the data; Wrote the paper.

Nadhira Al-Harrasi, Ruqaiya Al-Kindi: Conceived and designed the experiments; Performed the experiments.

Abdullah Al-Sulaimi: Performed the experiments; Analyzed and interpreted the data.

Hamad Al-Busaidi: Conceived and designed the experiments; Performed the experiments; Contributed reagents, materials, analysis tools or data.

Mohammed Al-Abri: Analyzed and interpreted the data; Wrote the paper.

### Funding statement

This work was supported by the Research Council of Oman (Grant No FRB/SQU/13/003) as part of Faculty Mentored Undergraduate Research Award Program.

### Competing interest statement

The authors declare no conflict of interest.

### Additional information

No additional information is available for this paper.

## Appendix A Supplementary data

S1 S1

S2 S2

S3 S3

S4 S4
