# Supplementary figures and images for: The role of urbanization in soil and groundwater contamination by heavy metals and pathogenic bacteria: A case study from Oman
Source: Heliyon. 2019 May 27;5(5):e01771. doi: 10.1016/j.heliyon.2019.e01771 (PMC6540334; doi:10.1016/j.heliyon.2019.e01771)

## Slide 1
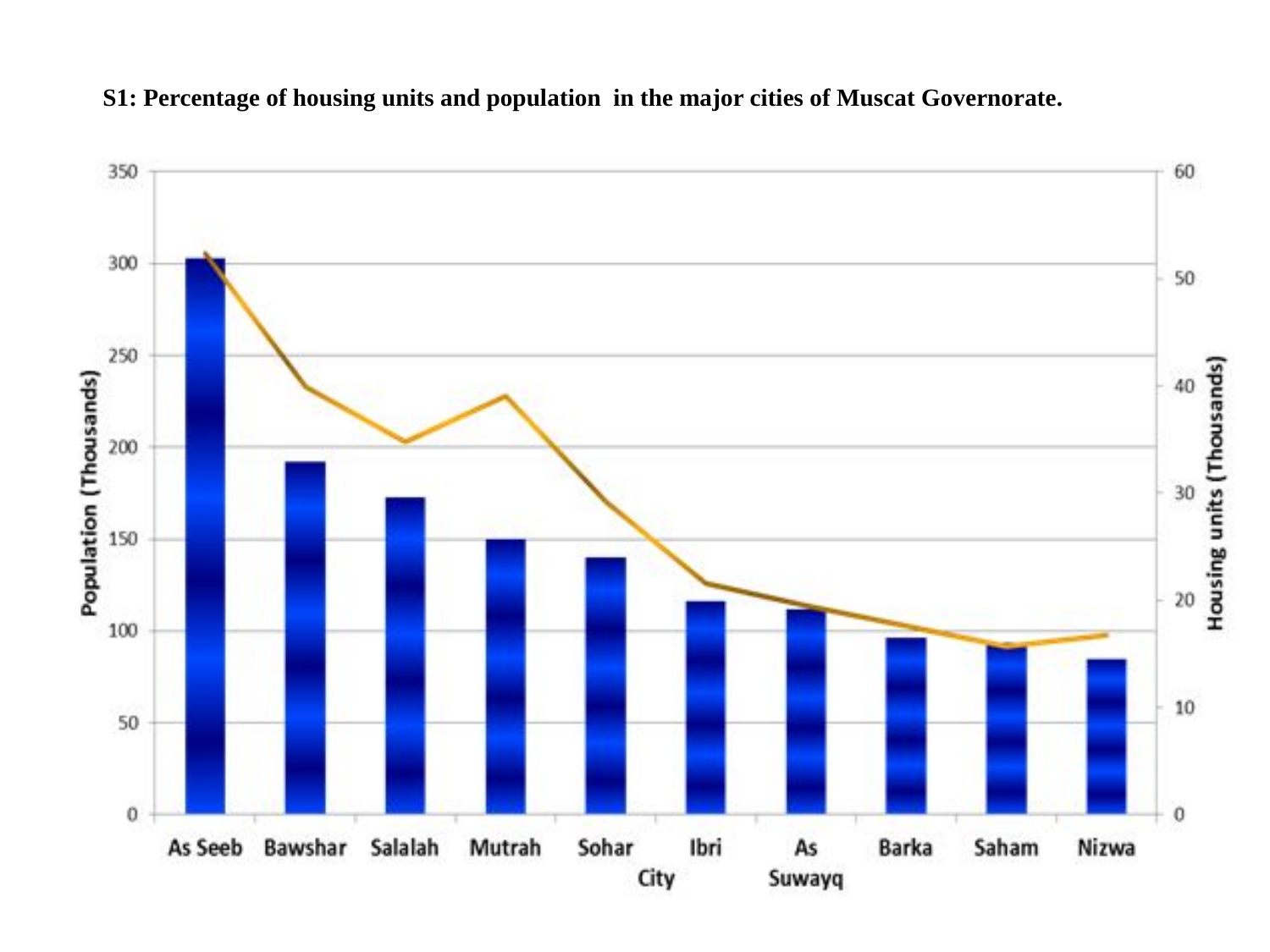

S1: Percentage of housing units and population in the major cities of Muscat Governorate.

Supplement: S1 [file mmc1.pptx]

## Slide 1
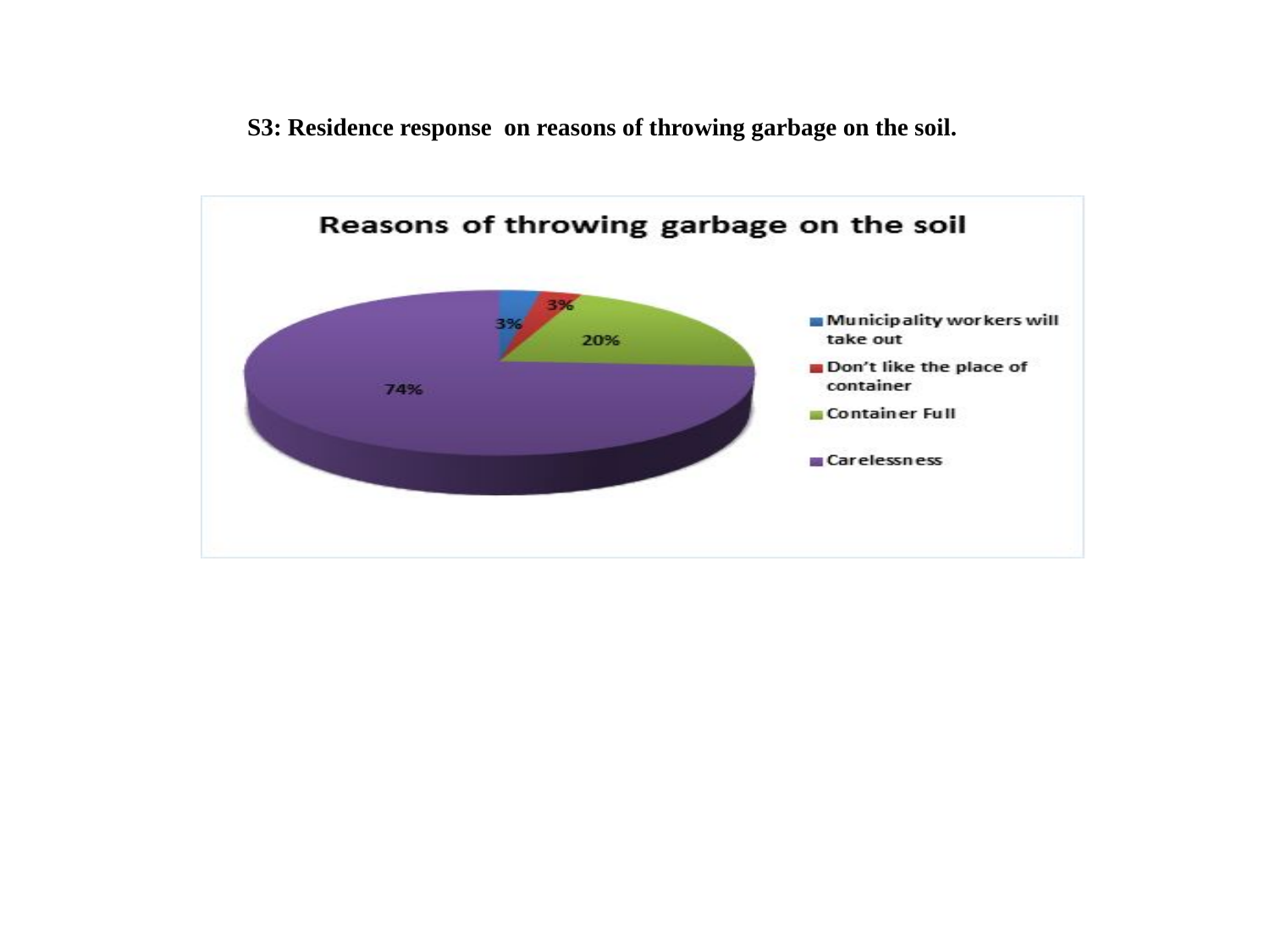

S3: Residence response on reasons of throwing garbage on the soil.

Supplement: S3 [file mmc3.pptx]
