# Supplementary material for: The role of urbanization in soil and groundwater contamination by heavy metals and pathogenic bacteria: A case study from Oman
Source: Heliyon. 2019 May 27;5(5):e01771. doi: 10.1016/j.heliyon.2019.e01771 (PMC6540334; doi:10.1016/j.heliyon.2019.e01771)
Supplement: S2 [file mmc2.docx]

S2: A questionnaire used to study the residents’ perceptions about urban gardening, municipal-waste disposal, and soil and water contamination.

| Sultan Qaboos University  College of Agricultural and Marine Sciences  Department of Soil, Water, and Agricultural Engineering | **Questionnaire code** |  |
| --- | --- | --- |
|  | **Date of interview**: …….. /……………../2013 | |
| **Objective of the study:**  This research aims to find out the most common soil manipulating anthropogenic activities that may have an impact on the characteristics of the urban soils of A’Seeb  The results of this research will provide precious information about urban soils in Oman. Thus, your cooperation by providing precise information is appreciated for the success of this research. All data gathered will remain strictly confidential and only will be used in the research. | | |

**Section 1: General information**

**--------------------------------------------------------------------------------------------------------------------------------------------------**

| 1. **Gender:** | |
| --- | --- |
| - Male | - Female |

| 1. **Age (in years): Please (✓)/العمر** | | | |
| --- | --- | --- | --- |
| - 18-22 | - 23-35 | - 36-50 | >50 |

| 1. **Where do you live?** | | |
| --- | --- | --- |
| - Al-Khurais | - Hail Al-Awamer | - North Al-Mawalih |

| 1. **Educational level** |
| --- |
| Did you go to school?   - Yes - No |
| If yes, what is your level of education? |
| - Primary school - Elementary - Secondary - Diploma - Undergraduate - Master degree - PhD |

**-------------------------------------------------------------------------------------------------------------------------------------Section 2: The use of urban soils for gardening and landscaping**

**---------------------------------------------------------------------------------------------------------------------------------------**

| 1. **How long have you lived here (in years)? Please (✓)** | | | |
| --- | --- | --- | --- |
| - 1-5 | - 6-10 | - 11-15 | - More than 15 |

| 1. **Are you interested in home gardening? Please (✓)** | | |
| --- | --- | --- |
| - Always | - Sometimes | - Never |

| 1. **What kind of crops do you grow? Please (✓)** |
| --- |
| - Edible |
| - Ornamental |
| - Both |
| - Nothing |

| 1. **If edible, do you consume them? Please (✓)** | | |
| --- | --- | --- |
| - Always | - Sometimes | - Never |

| 1. **Did you change your original soil by bringing another soil from another place? Please (✓)** | | |
| --- | --- | --- |
| - Yes | - No | - Not sure |

| 1. **If yes, how often you use to transport soil? Please (✓)** |
| --- |
| - Monthly |
| - Annually |
| - Other: ………………………………………… |

| 1. **Do you use fertilizers? Please (✓)** | | |
| --- | --- | --- |
| - Always | - Sometimes | - Never |

| 1. **If yes, what types of fertilizers do you use? Please (✓)** |
| --- |
| - Animal manure |
| - Green manure |
| - Chemical fertilizers |

| 1. **How often do you apply them? Please (✓)** | |
| --- | --- |
| - Often | - Rarely |

| 1. **Have you ever tested your soil? Please (✓)** | | |
| --- | --- | --- |
| - Yes | - No | - Not sure |

| 1. **Do you think that soil testing is important? Please (✓)** | |
| --- | --- |
| - Yes | - No |

| 1. **If yes, what types of soil testing is important? Please (✓)** |
| --- |
| - Soil fertility |
| - Soil contamination |
| - Soil stability |
| - Do not know |
| - Other: ………………………. |

| 1. **What soil contamination are you aware with and more concerned about in your garden? Please (✓)** |
| --- |
| - Salinity |
| - Heavy metal (e.g. lead, mercury, arsenic, cadmium) |
| - Biological hazard (e.g. pathogenic microbes) |
| - Organic chemicals (e.g. soil, pesticides) |
| - Do not know |
| - None |

| 1. **What source of water do you use for irrigation? Please (✓)** |
| --- |
| - Municipal supply water |
| - Well water |
| - Black water |
| - Greywater |

| 1. **How often do you irrigate your crop? Please (✓)** | | |
| --- | --- | --- |
| - Daily | - Day after day | - Weekly |

| 1. **In case of using black or greywater, why did you choose to directly irrigate your crop with this water? Please (✓)** |
| --- |
| - It is rich with nutrient, thus increase the yield |
| - To save some money instead of paying for bill of municipal water |
| - To use this water instead of let it go to the septic tank |

| 1. **How often do you irrigate your crop with black or greywater? Please (✓)** | | |
| --- | --- | --- |
| - Daily | - Once a week | - More than once a week |

| 1. **Have you ever heard about any regulation about the direct use of untreated black or greywater for irrigation in the city? Please (✓)** | |
| --- | --- |
| - Yes | - No |

| 1. **If yes, from whom? Please (✓)** |
| --- |
| - Neighbors |
| - Friends |
| - Ministry of Regional Municipalities and Water Resources (MRMWR) |
| - Other: …………………………… |

| 1. **Do you agree that the government should ban the direct use of untreated black or greywater? Please (✓)** |
| --- |
| - Strongly agree |
| - Agree |
| - Disagree |
| - Strongly disagree |

| 1. **Soil can host some pathogenic microbes (e.g. salmonella, E.coli, staphylococcus, etc.)? Please (✓)** |
| --- |
| - Strongly agree |
| - Agree |
| - Disagree |
| - Strongly disagree |
| - Don’t know |

| 1. **Untreated black or greywater could contaminate groundwater? Please (✓)** |
| --- |
| - Strongly agree |
| - Agree |
| - Disagree |
| - Strongly disagree |
| - Don’t know |

| 1. **Have you or your family ever felt that the irrigation with untreated black or greywater cause disease like abdomen pain, diarrhea or skin rash? Please (✓)** | |
| --- | --- |
| - Yes | - No |

| 1. **How do you think black or greywater can be used in a wise manner? Please (✓)** | | |
| --- | --- | --- |
| - Never use it | - Use it after treatment | - Not sure |

**-------------------------------------------------------------------------------------------------------------------------------------------------**

**Section 3: management and handling of municipal waste in urban soils community**

**--------------------------------------------------------------------------------------------------------------------------------------**

| 1. **How long the garbage container has been here? Please (✓)** |
| --- |
| - <2 months |
| - 2-6 months |
| - 1-5 years |
| - 6-10 years |
| - >10 years |
| - Not sure |
| - Do not know |

| 1. **Have you ever seen that people or municipality workers moved the garbage container its place? Please (✓)** | |
| --- | --- |
| - Yes | - No |

| 1. **Why do you think people used to throw their garbage next to the garbage container rather than throwing them inside? Please (✓)** |
| --- |
| - The municipality people will come and take it |
| - I don’t like the place of the garbage container |
| - The container is usually full |
| - Just careless |
| - Other: ……………… |

| 1. **Who is in your house is usually throwing the garbage? Please (✓)** |
| --- |
| - Children |
| - Housemaid |
| - Gardener |
| - Myself |

| 1. **Throwing garbage materials in soil could increase the propagation of pathogenic microbes (e.g. salmonella, E.Coli, staphylococcus, etc.)? Please (✓)** |
| --- |
| - Strongly agree |
| - Agree |
| - Disagree |
| - Strongly disagree |
| - Don’t know |

| 1. **Chemicals from garbage material can deeply transport in the soil? Please (✓)** |
| --- |
| - Strongly agree |
| - Agree |
| - Disagree |
| - Strongly disagree |
| - Don’t know |

| 1. **Throwing garbage material in soil could have an impact on the morphological features of soils (e.g. color, structure, adds artifacts, etc.)? Please (✓)** |
| --- |
| - No impact |
| - Small impact |
| - Moderate impact |
| - Big impact |
| - No idea |

| 1. **Throwing garbage material in soil could lead to surface or groundwater contamination? Please (✓)** |
| --- |
| - Strongly agree |
| - Agree |
| - Disagree |
| - Strongly disagree |
| - Don’t know |

| 1. **Do you concern about the spreading of contamination or even the possible health hazard due to throwing garbage in the soil? Please (✓)** | |
| --- | --- |
| - Yes | - No |

| 1. **Have you ever heard about selective waste disposal system? Please (✓)** | |
| --- | --- |
| - Yes | - No |

| 1. **If not, (the selective system in waste disposal works to perform a separate collection of garbage material –e.g. selective separation of papers, glass, food waste, metals, chemicals, etc.-). Now, do you agree that this system would be an applicable and feasible solution to reduce any possible impact in the urban environment? Please (✓)** |
| --- |
| - Strongly agree |
| - Agree |
| - Disagree |
| - Strongly disagree |
| - Don’t know |

Thank you for your corporation
