# Supplementary material for: The role of urbanization in soil and groundwater contamination by heavy metals and pathogenic bacteria: A case study from Oman
Source: Heliyon. 2019 May 27;5(5):e01771. doi: 10.1016/j.heliyon.2019.e01771 (PMC6540334; doi:10.1016/j.heliyon.2019.e01771)
Supplement: S4 [file mmc4.docx]

**S4: Selected soil profile descriptions of the study area.**

**Profile name: Profile 1**

Soil Classification: USDA: Typic Torriorthent

Location: North Al-Hail 23° 39' 11.9" N; 58° 13' 08.1’’

Profile description:

| A | 0-21 cm | Dark brown (10 YR 3/3, moist); loamy sand; massive; fibrous roots; 1% gravel; strongly effervescent; none saline; slightly alkaline; diffuse wavy boundary |
| --- | --- | --- |
| C1 | 21-66 cm | Brown (10 YR 4/3, moist); loamy sand; massive; fibrous roots; 1% gravel; slightly effervescent; none saline; slightly alkaline; diffuse wavy boundary |
| C2 | 66-78 cm | Brown (10 YR 4/3, moist); sandy loam; massive; fibrous roots; 1% gravel; slightly effervescent; none saline; slightly alkaline; diffuse smooth boundary |
| C3 | 78-99 cm | Brown (10Y R 5/3, moist); loam; massive; fibrous roots; 45% gravel; strongly effervescent; none saline; slightly alkaline; diffuse smooth boundary |
| C4 | >99 | Brown (10Y R 5/3, moist); loam; massive; fibrous roots; 1% gravel; very slightly effervescent; none saline; slightly alkaline; diffuse smooth boundary |

**Profile name: Profile 2**

Soil Classification: USDA: Haplic Torriarent

Location: North Al-Hail 23° 39' 14.2" N; 58° 13' 11.2’’

Profile description:

| A | 0-24 cm | Dark brown (7.5 YR 3/2, moist); loamy sand; massive; no roots; 25% gravel; very slightly effervescent; very slightly saline; slightly alkaline; artifacts (i.e. ceramic and glass) gradual smooth boundary |
| --- | --- | --- |
| C1 | 24-43 cm | Dark brown (7.5 YR 3/2, moist); loamy sand; massive; no roots; 40% gravel; very slightly effervescent; very slightly saline; slightly alkaline; gradual smooth boundary |
| C2 | 43-134 cm | Dark brown (7.5 YR 3/2, moist); loamy sand; massive; no roots; 1% gravel; very slightly effervescent; slightly saline; slightly alkaline; abrupt smooth boundary |

**Profile name: Profile 3**

Soil Classification: USDA: Typic Torrifluvent

Location: North Al-Hail 23° 41' 6.68" N; 57° 54' 43.37’’

Profile description:

| A | 0-15 cm | Dark yellowish brown (10 YR 4/4, moist); silt loam; massive; no roots; 40% gravel; very slightly effervescent; none saline; moderately alkaline; diffuse smooth boundary |
| --- | --- | --- |
| C1 | 15-35 cm | Dark yellowish brown (10 YR 4/4, moist); silt loam; massive; no roots; 15% gravel; very slightly effervescent; none saline; moderately alkaline; diffuse smooth boundary |
| C2 | 35-71 cm | Dark yellowish brown (10 YR 4/4, moist); silt loam; massive; no roots; 15% gravel; very slightly effervescent; none saline; moderately alkaline; diffuse smooth boundary |
| C3 | 71-133 cm | Dark yellowish brown (10 YR 4/4, moist); silt loam; massive; no roots; 30% gravel; very slightly effervescent; none saline; strongly alkaline; diffuse smooth boundary |

**Profile name: Profile 4**

Soil Classification: USDA: Typic Haplosalids

Location: North Al-Hail 23° 38' 16.4" N; 58° 14.4' 19’’

Profile description:

| A | 0-35cm | Dark yellowish brown (10 YR 3/4, moist); loamy sand; massive; no roots; 77% gravel; very slightly effervescent; moderately saline; slightly alkaline; artifacts (plastic containers and plastic bags); abrupt wavy boundary |
| --- | --- | --- |
| B_z_ | 35-62 cm | Dark yellowish brown (10 YR 3/4, moist); loamy sand; massive; no roots; 47% gravel; very slightly effervescent; moderately saline; neutral; abrupt smooth boundary |
| C1 | 62-80 cm | Dark yellowish brown (10 YR 3/4, moist); sandy loam; massive; no roots; 8% gravel; very slightly effervescent; moderately saline; neutral; diffuse smooth boundary |
| C2 | 80-130 cm | Dark yellowish brown (10 YR 3/4, moist); sandy loam; massive; no roots; 5% gravel; very slightly effervescent; strongly saline; neutral; diffuse smooth boundary |
